# Down-Regulation of Human Enteric Antimicrobial Peptides by NOD2 during Differentiation of the Paneth Cell Lineage

**DOI:** 10.1038/srep08383

**Published:** 2015-02-11

**Authors:** Gao Tan, Run-hua Li, Chen Li, Fang Wu, Xin-mei Zhao, Jia-yi Ma, Shan Lei, Wen-di Zhang, Fa-chao Zhi

**Affiliations:** 1 Guangdong Provincial Key Laboratory of Gastroenterology, Department of Gastroenterology, Nanfang Hospital, Southern Medical University, Guangzhou 510515, China

## Abstract

Ileal Crohn's disease (CD) arising from the alteration of intestinal homeostasis is characterized by two features, namely a decrease in Paneth cell-produced antimicrobial peptides that play a key role in maintaining this balance and an increase in NOD2, an intracellular sensor. Although mutations in NOD2 are highly correlated with the incidence of CD, the physiological role of NOD2 in intestinal immunity remains elusive. Here, we show that NOD2 can down-regulate the expression of human enteric antimicrobial peptides during differentiation of the Paneth cell lineage. This finding, which links the decrease of human enteric antimicrobial peptides to increased NOD2 in ileal CD patients, provides a new view into the pathogenesis of ileal CD.

Crohn's disease (CD), the main clinical phenotype of inflammatory bowel disease (IBD)^1^, is a chronic, relapsing inflammatory disorder^2^. Although CD can occur anywhere in the gastrointestinal tract, it primarily affects the terminal ileum where as many as 75% of CD patients have inflammation^3^. The terminal ileum is characterized by two relevant features: the greatest number of Paneth cells^4^ that are generally absent from the colon and rectum, except in IBD^5^, and the highest microbial density, which is low in healthy proximal small intestine^3^,^6^. Human Paneth cells serve as a key arm of innate mucosal immunity to maintain the intestinal homeostasis between a host and its colonizing microbes by secreting antimicrobial peptides^7^,^8^. These antimicrobial peptides are composed predominantly of human enteric α-defensin 5 and 6 (HD5 and HD6) as well as lysozyme and secretory phospholipase A2 (sPLA2), to a lesser extent^9^. These peptides not only have a strong antibacterial function against Gram-positive and Gram-negative bacteria, but they also have activity against viruses, fungi and protozoa^7^,^10^,^11^,^12^. Their antimicrobial activities contribute to their roles in intestinal innate immunity. In addition, human Paneth cells express NOD2^13^, a member of the nucleotide-binding oligomerization domain-leucine-rich repeat (NOD-LRR) proteins^14^, and the Paneth cell expression of NOD2 is increased in CD patients^13^. Although mutations in NOD2 are highly correlated with a diminished expression of human enteric α-defensin^15^ and the incidence of CD^16^,^17^, the physiological role of NOD2 in intestinal immunity remains elusive.

The purpose of this study was to determine whether NOD2 may regulate the expression of human enteric antimicrobial peptides. For this purpose, we should choose a suitable cell line because human Paneth cells do not survive under *in* *vitro* culture conditions^13^,^18^. Because Caco2 intestinal epithelial cells can display characteristics of small intestinal epithelial differentiation *in* *vitro*^19^,^20^ and constitutively express the NOD2 gene^13^, they are suitable for *in* *vitro* studies to investigate the physiological role of NOD2 in specialized intestinal epithelial cells such as Paneth cells.

## Results

### Activation of FGFR-3-mediated signaling induces in vitro differentiation of Caco2 cells along the Paneth cell lineage

**Figure 1:**
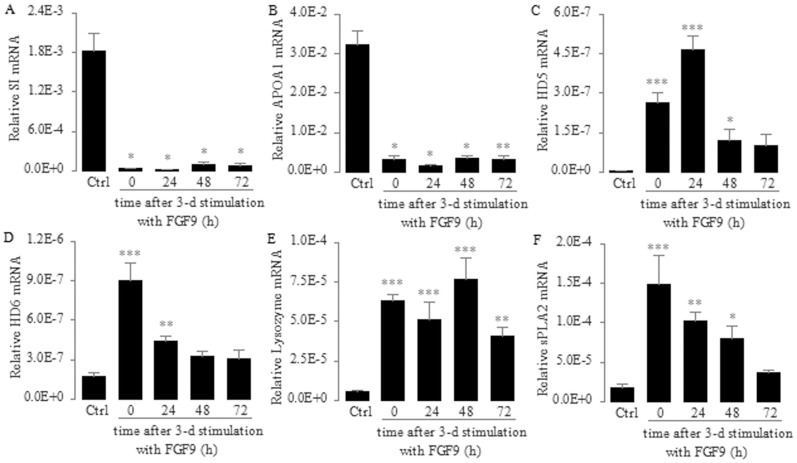
Effect of FGFR-3-mediated signaling on the expression of intestinal lineage differentiation markers in Caco2 cells. Enterocyte markers: sucrase-isomaltase (SI; A) and apolipoprotein A-1 (APOA1; B). Paneth cell markers: human α-defensin 5 (HD5; C), human α-defensin 6 (HD6; D), Lysozyme (E) and secretory phospholipase A2 (sPLA2; F). Caco2 cells were treated with FGF9 (10 ng/ml) daily for 3 consecutive days. After that, the cells were not treated with FGF9 but their medium was changed every 24 h. Total RNA was isolated and mRNA levels were determined using real-time PCR and normalized to 18 S rRNA at the indicated time points after the 3-day stimulation with FGF9. Control (Ctrl) cells were not treated with FGF9. Data are shown as the mean ± SD of three independent experiments; *P < 0.05, **P < 0.01, ***P < 0.001 vs. control groups.

Although Caco2 cells can spontaneously differentiate along the enterocyte lineage *in* *vitro*^21^,^22^, they also express abundant FGFR-3^23^, which is a critical regulator of Paneth cell differentiation during mouse gut development^24^. Therefore, we treated Caco2 cells with FGF9, a high affinity ligand for FGFR-3^25^, and determined whether the activation of FGFR-3-mediated signaling induces *in vitro* differentiation of Caco2 cells along the Paneth cell lineage. We found that the mRNA expression of *SI* and *APOA1*, which encode two enterocyte differentiation markers^25^, was greatly decreased. These significant decreases were sustained for at least 72 h after a consecutive 3-day treatment with FGF9 (Fig. 1A and 1B), suggesting that the differentiation of Caco2 cells along the enterocyte lineage is suppressed and that this differential inhibition is stable. In contrast, we found that the mRNA expression of *HD5*, *HD6*, *lysozyme* and *sPLA2,* which encode four Paneth cell differentiation markers, was greatly increased after a consecutive 3-day treatment with FGF9 (Fig. 1C–1F). In addition, we found that these significant increases were sustained for at least 24 h after a consecutive 3-day treatment with FGF9 (Fig. 1C–1F). These results indicate that the activation of FGFR-3-mediated signaling can induce the *in vitro* differentiation of Caco2 cells along the Paneth cell lineage and maintain this induction of differentiation for a period of time.

### NOD2 signaling down-regulates the expression of human enteric antimicrobial peptides during differentiation of the Paneth cell lineage

**Figure 2:**
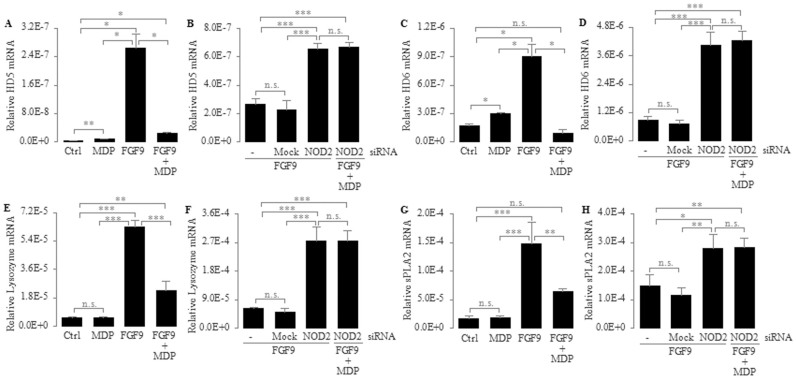
Differential regulation of mRNA expression of *HD5* (A, B), *HD6* (C, D), *Lysozyme* (E, F) and *sPLA2* (G, H) by different stimuli in Caco2 cells. (A, C, E, G) Caco2 Cells were treated with MDP (10 μg/ml), FGF9 (10 ng/ml) or FGF9 (10 ng/ml) plus MDP (10 μg/ml) daily for 3 consecutive days. (B, D, F, H) Caco2 cells were transfected with transfection reagent only (mock) or NOD2 siRNA (50 nM) for 6 h, then incubated with normal growth medium for an additional 18 h. Subsequently, these cells were treated with FGF9 (10 ng/ml) or FGF9 (10 ng/ml) plus MDP (10 μg/ml) daily for 3 consecutive days. Total RNA was isolated and mRNA levels were determined using real-time PCR and normalized to 18 S rRNA. Control (Ctrl) cells were not treated. Data are shown as the mean ± SD of three independent experiments; n.s., not significant, *P < 0.05, **P < 0.01, ***P < 0.001.

To determine the effect of the NOD2 gene on the expression of human enteric antimicrobial peptides, we first asked whether NOD2 regulates FGF9-induced expression of human enteric antimicrobial peptides during differentiation of the Paneth cell lineage. We utilized FGF9 with or without MDP, an agonist for NOD2^26^, to stimulate Caco2 cells for 3 consecutive days and examined the mRNA expression of *HD5*, *HD6*, *lysozyme* and *sPLA2* using real-time PCR. We found that the mRNA expression of *HD5*, *HD6*, *lysozyme* and *sPLA2* was decreased approximately 10.6-, 9.6-, 2.7- and 2.3-fold, respectively, in Caco2 cells treated with MDP plus FGF9 compared with FGF9 only (Fig. 2A, 2C, 2E and 2G). This result indicates that MDP-NOD2 signaling can down-regulate the expression of human enteric antimicrobial peptides, especially enteric α-defensin, during differentiation of the Paneth cell lineage.

**Figure 3:**
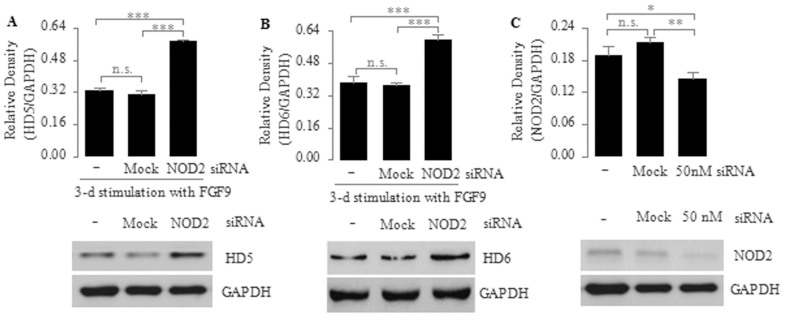
Down-regulation of FGF9-mediated protein expression of *HD5* (A) and *HD6* (B) by NOD2 in Caco2 cells. (A, B) Caco2 cells were transfected with transfection reagent only (mock) or NOD2 siRNA (50 nM) for 6 h, then incubated with normal growth medium for an additional 18 h. Subsequently, these cells were treated with FGF9 (10 ng/ml) daily for 3 consecutive days. Whole-cell extracts were analyzed for HD5 by immunoblotting. (C) Transfection efficiency was tested after a 72-h transfection with siRNA via immunoblotting. Top, quantitative analysis of proteins; bottom, representative immunoblot images. Data are shown as the mean ± SD of three independent experiments; n.s., not significant, *P < 0.05; **P < 0.01; ***P < 0.001.

To further substantiate the role of NOD2 in regulating FGF9-induced expression of human enteric antimicrobial peptides, we transfected Caco2 cells with a NOD2-specific siRNA, followed by a consecutive 3-day stimulation with FGF9 or FGF9 plus MDP. We found that the mRNA expression of *HD5*, *HD6*, *lysozyme* and *sPLA2* was significantly higher in Caco2 cells transfected with NOD2-siRNA than in untransfected or mock transfected cells (Fig. 2B, 2D, 2F and 2H), suggesting that NOD2 signaling can down-regulate the FGF9-induced expression of human enteric antimicrobial peptides. Consistent with this result, the protein expression of *HD5* and *HD6* was significantly increased in FGF9-stimulated NOD2-siRNA-transfected cells compared with untransfected or mock transfected cells (Fig. 3A and 3B), thus further confirming that NOD2 signaling can down-regulate the expression of human enteric antimicrobial peptides during differentiation of the Paneth cell lineage; however, we found no significant differences in the mRNA expression of *HD5*, *HD6*, *lysozyme* and *sPLA2* between FGF9-stimulated NOD2-siRNA-transfected cells and NOD2-siRNA transfectants stimulated with FGF9 plus MDP (Fig. 2B, 2D, 2F and 2H), suggesting that the down-regulated mRNA expression of human enteric antimicrobial peptides is indeed mediated by MDP via NOD2. In addition, we found that NOD2 protein expression was significantly lower in NOD2-siRNA transfected cells than in mock or untransfected cells (Fig. 3C), thus confirming the efficiency of NOD2-knockdown via siRNA.

### NOD2 itself differentially regulates the expression of human enteric antimicrobial peptides

We next determined whether NOD2 itself can affect the expression of human enteric antimicrobial peptides. We treated Caco2 cells with the NOD2 agonist MDP and then determined the mRNA expression of *HD5*, *HD6*, *lysozyme* and *sPLA2* using real-time PCR. We found that the mRNA expression of *HD5* and *HD6* was increased approximately 2.8- and 1.7-fold, respectively, in Caco2 cells after a consecutive 3-day treatment with MDP compared with untreated control (Ctrl) cells (Fig. 2A and 2C). This result demonstrates that NOD2 itself can slightly up-regulate the expression of human enteric α-defensin 5 and 6, which is consistent with the report showing the decreased expression of Paneth cell α-defensins in NOD2-knockout mice^27^; however, the mRNA expression of *lysozyme* and *sPLA2* was not significantly different between MDP-treated cells and untreated control (Ctrl) cells (Fig. 2E and 2G), suggesting that NOD2 itself does not affect the expression of *lysozyme* and *sPLA2*.

### FGF9 does not regulate NOD2 expression during differentiation of the Paneth cell lineage

**Figure 4:**
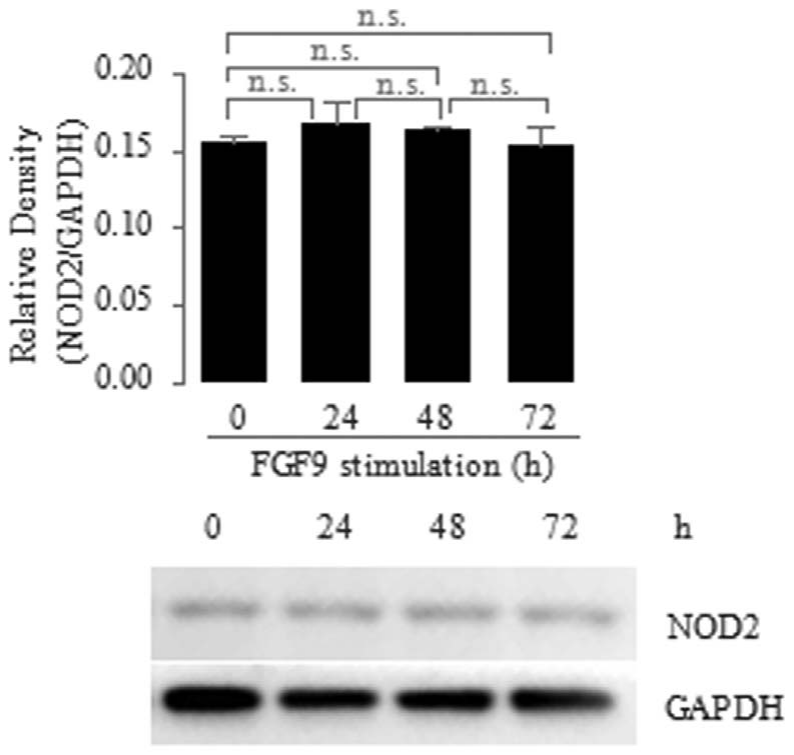
No Effect of FGF9 on protein expression of *NOD2* in Caco2 cells. Caco2 cells were treated with FGF9 (10 ng/ml) for the indicated time points, after which whole-cell extracts were prepared and analyzed for NOD2 via immunoblotting. Top, quantitative analysis of proteins; bottom, the representative immunoblotting image from three independent experiments. Data are shown as the mean ± SD of three independent experiments; n.s., not significant.

Finally, we determined whether FGF9 treatment affected NOD2 expression during differentiation of the Paneth cell lineage. We treated Caco2 cells with FGF9 and determined the protein expression of *NOD2* via immunoblotting at different times of induction of cell differentiation. We found that *NOD2* protein expression was not significantly different between FGF9-treated cells and untreated control (Ctrl) cells (Fig. 4). This result indicates that FGF9 does not regulate NOD2 expression during differentiation of the Paneth cell lineage.

## Discussion

This study assessed whether NOD2 can regulate the expression of human enteric antimicrobial peptides. Our data in Caco2 cells show that NOD2 can down-regulate the expression of human enteric antimicrobial peptides during differentiation of the Paneth cell lineage. We found that the Caco2 cells treated with FGF9 can be induced into differentiation along the Paneth cell lineage (Fig. 1). During this induction of cell differentiation, the mRNA expression of human enteric antimicrobial peptides (HD5, HD6, Lysozyme and sPLA2) was decreased in the presence of the stimulation of NOD2 agonist MDP but was increased when NOD2 was knocked down by a NOD2-specific siRNA (Fig. 2). In addition, we found that the protein levels of *HD5* and *HD6*, the main constituents of human enteric antimicrobial peptides, were increased in FGF9-stimulated NOD2-siRNA-transfected cells compared with untransfected cells (Fig. 3), thus further confirming that NOD2 can down-regulate the expression of human enteric antimicrobial peptides during differentiation of the Paneth cell lineage.

Whether the molecular mechanism of NOD2-mediated down-regulation of expression of human enteric antimicrobial peptides is dependent on or independent of the ability of NOD2 to recruit and activate downstream cofactor RICK or others remains unknown; however, previous findings show that NOD2 activated by MDP can bind and activate RICK, a caspase recruitment domain (CARD)-containing serine/threonine kinase^28^,^29^. In conjunction with our own findings, we speculate that FGFR-3-NOD2 signaling may recruit a kind of signaling molecules different from RICK to inhibit FGFR-3-induced expression of human enteric antimicrobial peptides. We hypothesize that this signaling pathway may enhance the inhibition efficiency through recruiting more of that kind of signaling molecules when NOD2 is simultaneously activated upon MDP stimulation.

Although NOD2 can down-regulate FGF9-induced expression of *HD5*, *HD6*, *Lysozyme* and *sPLA2*, NOD2 itself can up-regulate the expression of *HD5* and *HD6*. We found that the mRNA expression of *HD5* and *HD6* was increased in MDP-stimulated cells compared with untreated control cells (Fig. 2). This result is in line with the previous report showing the decreased expression of Paneth cell α-defensins in NOD2-knockout mice^27^. Because FGF9 does not affect NOD2 expression during differentiation of the Paneth cell lineage (Fig. 4), it is extremely interesting to explore the mechanism by which NOD2 dually regulates the expression of human enteric α-defensins under the different types of NOD2 stimuli.

In this study, we used Caco2 cells, serving as a functional modal, to investigate whether NOD2 regulates the expression of human enteric antimicrobial peptides in the Paneth cell lineage. This cell line is suitable because primary Paneth cells do not survive *in* *vitro*^13^,^18^; however, Caco2 cells can display characteristics of small intestinal epithelial differentiation *in* *vitro*^21^,^22^, suggesting that they maintain intestinal stem cell functions. In addition, they constitutively express the NOD2 gene^13^ and also express abundant FGFR-3^25^, which is a critical regulator of Paneth cell differentiation during gut development^24^. Finally, we found that Caco2 cells activated by FGFR-3-mediated signaling for 3 consecutive days express Paneth cell lineage-specific genes. Thus, Caco2 cells are suitable for this *in* *vitro* study to investigate the role of the NOD2 protein in the Paneth cell lineage.

**Figure 5:**
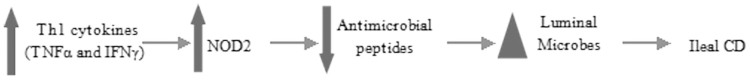
Schematic diagram of the proposed hypothesis for the role of NOD2 in the pathogenesis of ileal CD. Th1 cytokines such as TNFα and IFNγ up-regulate NOD2 expression. A large increase in NOD2 levels causes a large decrease in antimicrobial peptides including HD5, HD6, lysozyme and sPLA2, leading to weakened mucosal defenses,and alterations in the luminal microbes. These changes lead to the destruction of intestinal homeostasis, which then induces and/or sustains inflammation in susceptible individuals. This pathogenesis ultimately results in ileal CD. Abbreviations: NOD2, nucleotide-binding oligomerization domain 2; CD, Crohn's disease; TNFα, tumor necrosis factor α; IFNγ, interferon-γ; HD5, human α-defensin 5; HD6, human α-defensin 6; sPLA2, secretory phospholipase A2.

In summary, our results indicate that NOD2 can down-regulate the expression of human enteric antimicrobial peptides during differentiation of the Paneth cell lineage. In light of NOD2 over-expression in CD patients^13^, our data provide a plausible explanation for the diminished levels of human enteric antimicrobial peptides in ileal CD patients. This finding is significant because a strongly advocated view is that the ineffective bacterial clearance that results from the reduced expression of human enteric antimicrobial peptides^27^ induces and sustains the abnormal adaptive immune responses observed in CD patients^30^,^31^. In addition, T helper 1 (Th1) cytokines such as factor α (TNFα) and interferon-γ (IFNγ) can up-regulate NOD2 expression in intestinal epithelial cells^32^,^33^,^34^. Based on these findings, a hypothesis for the role of NOD2 in the pathogenesis of CD is proposed (Fig. 5) in which NOD2 constitutes a critical link between the innate and adaptive immunity in the intestinal tract. Indeed, anti-TNFα, anti-IFNγ or anti-interleukin-12 administration is an effective therapeutic strategy in CD^35^,^36^,^37^,^38^,^39^,^40^,^41^, although none are a permanent cure for CD. As discussed above, we speculate that if these cytokine-based therapies work by interrupting the over-expression of NOD2, the most effective therapy for CD patients will be directed at antagonism of NOD2-mediated inhibition of human antimicrobial peptides.

## Methods

### Cell culture and stimulation

Caco2 cells (ATCC) were cultured in Dulbecco's modified Eagle medium (HyClone) supplemented with 20% fetal calf serum (HyClone), 2 mM L-glutamine, 100 U/ml Penicillin, and 100 μg/ml streptomycin at 37°C in a humidified atmosphere with 5% CO2. The cells were used between passages 15 and 30. For all of the experiments, to better mimic the steric conditions existing in the intestine *in vivo*, the cells were plated at a subconfluent cell density onto 6-well Millicell hanging filter inserts (3 μm pore size, Polyethylene Terephthalate, Millipore) that allow free access of media to their apical and basolateral sides. Media were changed every 24 h. To determine the role of NOD2, fibroblast growth factor 9 (FGF9) (10 ng/ml; R&D Systems), a high affinity ligand for the fibroblast growth factor receptor-3 (FGFR-3); muramyl dipeptide (MDP) (10 μg/ml; InvivoGen), an agonist for intracellular NOD2; and FGF9 (10 ng/ml) plus MDP (10 μg/ml) were added to both sides of the inserts daily starting at 24 h post-plating and ending at 72 h post-plating.

### SiRNA

After 24-h culture with antibiotic-free normal growth medium containing 20% fetal calf serum, Caco2 cells that were approximately 60% confluent were transfected with the NOD2 siRNA (50 nM; Santa Cruz Biotechnology) and Transfection Reagent (Santa Cruz Biotechnology) mixture or Transfection Reagent only (Mock) for 6 h, then incubated with normal growth medium for an additional 18 h. Subsequently, these cells were stimulated upon the addition of FGF9 (10 ng/ml) or FGF9 (10 ng/ml) plus MDP (10 μg/ml) daily for 3 days. After that, total RNA was isolated and analyzed using real-time PCR. Whole-cell extracts were prepared and analyzed via immunoblotting. The transfection efficiency was tested after a 72-h transfection with siRNA via immunoblotting.

### Real-time quantitative RT-PCR

Total cellular RNA was isolated using the RNAiso Plus Kit (Takara) and then cDNA synthesis was performed using the PrimeScript RT reagent Kit with gDNA Eraser (Takara) to eliminate genomic DNA contamination. Real-time PCR was performed in triplicate using the LightCycler 480 System (Roche). Each 20 μl PCR reaction contained 5 μl of cDNA corresponding to 25 ng of RNA as a template, 0.5 μM of each primer (table 1), and 1 × LightCycler 480 SYBR Green I Master (Roche). The samples were loaded into the LightCycler 480 Multiwell Plate 96 (Roche) and incubated for an initial denaturation at 95°C for 10 min followed by 45 cycles, with each cycle consisting of 95°C for 10 s, a “touchdown” of −1°C/cycle from 65°C 60°C for 20 s, followed by 72°C for 20 s. Relative mRNA levels were calculated according the 2^−ΔCT^ method, using 18 S rRNA as the reference and internal standard.

### Immunoblotting

The cells were lysed for 30 min on ice in RIPA lysis buffer (10 mM Tris (pH 8.0), 150 mM NaCl, 1% Nonidet P-40, 0.1% SDS, and 0.5% deoxycholate, supplemented with the protease inhibitor PMSF. After being centrifuged at 14,000 × *g* for 30 min at 4°C the supernatants were collected. SDS-polyacrylamide gel electrophoresis and western blotting were performed in accordance with standard protocols. Monoclonal mouse anti-HD5 (Millipore), anti-HD6 (Biorbyt) and polyclonal goat anti-NOD2 (Santa Cruz Biotechnology) were diluted at 1:1000, 1:1000 and 1:200, respectively. Monoclonal rabbit anti-GAPDH (Cell Signaling Technology) was diluted at 1:1000. Secondary antibodies were all diluted at 1:4000. Image J software was used to quantify and analyze the density of the protein bands.

### Statistical Analysis

The results are shown as the mean ± standard deviation. Statistical significance was determined by one-way analysis of variance with Tukey's multiple comparisons under equal variances or with Dunnett T3's multiple comparisons under unequal variances; a value of *P* < 0.05 was considered statistically significant.

## Author Contributions

G.T. designed the studies, performed the experiments, wrote the manuscript and prepared the table and figures. F.Z. conceived the studies and reviewed the manuscript. R.L., C.L., X.Z., F.W., J.M., S.L. and W.Z. reviewed the manuscript.

## Figures and Tables

**Table 1:** Real-time quantitative RT-PCR primer sequences

Gene	Sense	Antisense
*APOA1*	AGC TTG CTG AAG GTG GAG GT	ATC GAG TGA AGG ACC TGG C
*SI*	ACC CAA TCG TTT CCG GTT CA	GGG TTT TGG GCA ACC TTC AC
*HD5*	GCC ATC CTT GCT GCC ATT C	AGA TTT CAC ACA CCC CGG AGA
*HD6*	CCT CAC CAT CCT CAC TGC TGT TC	CCA TGA CAG TGC AGG TCC CAT A
*Lysozyme*	AAA ACC CCA GGA GCA GTT AAT	CAA CCC TCT TTG CAC AAG CT
*sPLA2*	TGA CGA CAG GAA AGG AAG CCG CAC	AGG GAA GAG GGG ACT CAG CAA CGA G
*18 S rRNA*	TTT GTT GGT TTT CGG AAC TGA	CGT TTA TGG TCG GAA CTA CGA

APOA1: apolipoprotein A-1; SI: sucrase-isomaltase; HD5: human α-defensin 5; HD6: human α-defensin 6; sPLA2: secretory phospholipase A2.
